# Supplementary material for: Secretome Analysis of Arabidopsis–Trichoderma atroviride Interaction Unveils New Roles for the Plant Glutamate:Glyoxylate Aminotransferase GGAT1 in Plant Growth Induced by the Fungus and Resistance against Botrytis cinerea
Source: Int J Mol Sci. 2021 Jun 24;22(13):6804. doi: 10.3390/ijms22136804 (PMC8268252; doi:10.3390/ijms22136804)
Supplement: Supplementary file 1 [file ijms-22-06804-s001.zip › ijms-1240257-supplementary.pdf]

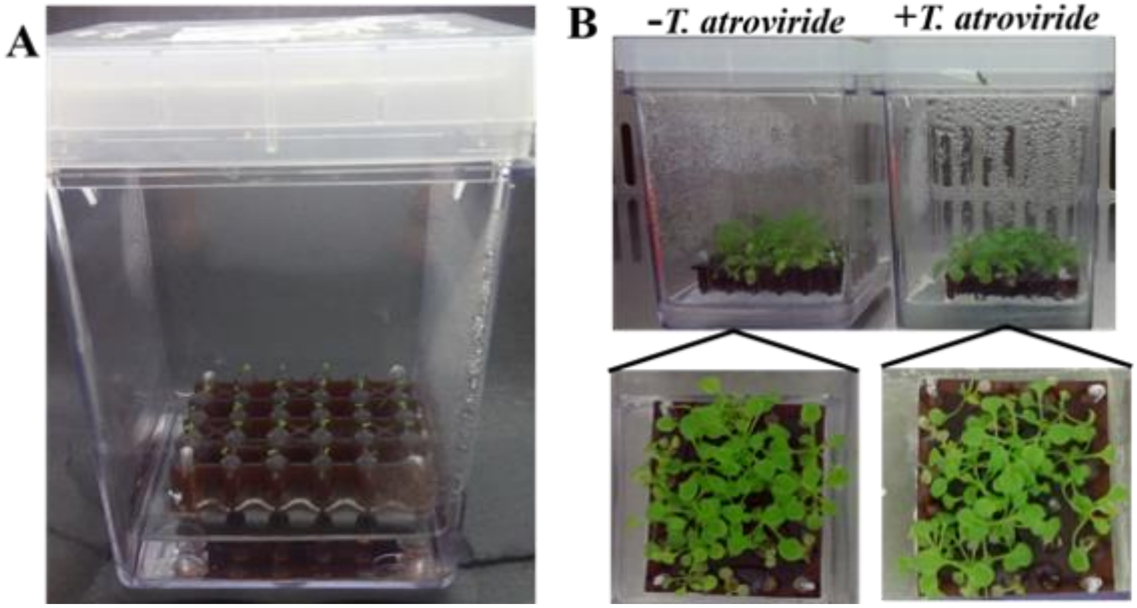

Figure S1. Representative pictures of *Arabidopsis* seedlings growing alone or interacting with *T. atroviride* in a semi-hydroponic system. (**A**) Semi-hydroponic system with 2-day-old *Arabidopsis* seedlings transplanted on MS agar over a perforated plastic stand in magenta® boxes. MS medium was added to each semi-hydroponic system, which was replaced when *T. atroviride* was inoculated (see material and methods). (**B**) 3-week-old *Arabidopsis* seedlings at 24 hpi with *T. atroviride* aseptically growing in the semi-hydroponic system.

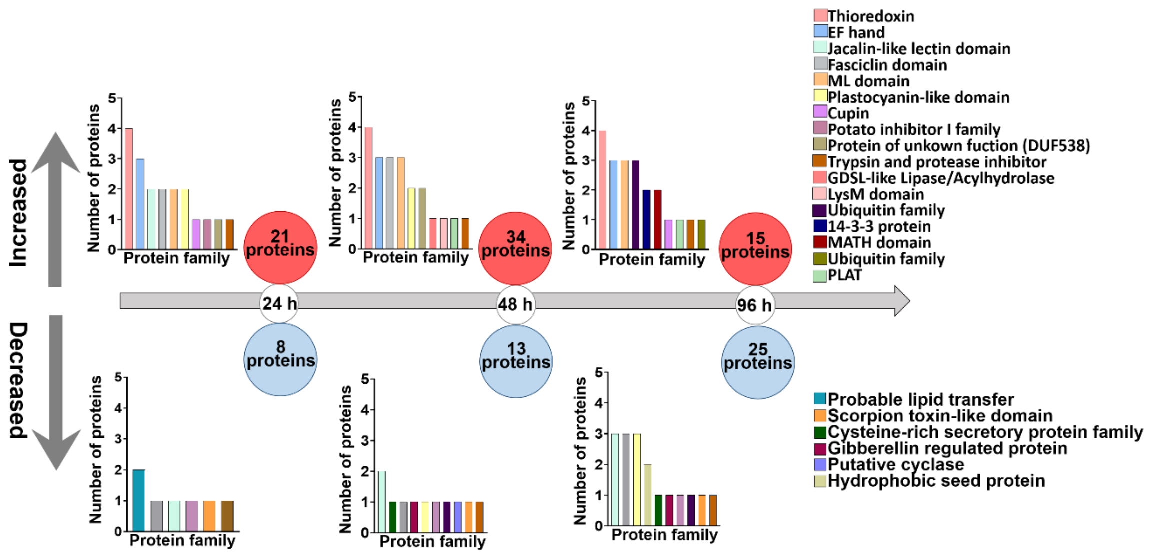

Figure S2. Protein family classification of *Arabidopsis* proteins secreted in response to *T. atroviride* at 24, 48 and 96 h of co-culture. Graphic representation for the *T. atroviride* proteins differentially modulated (increased or decreased) according to protein family classification predicted by HMMER server [93]. Only proteins that were not predicted as enzymes according to EC nomenclature system are shown.

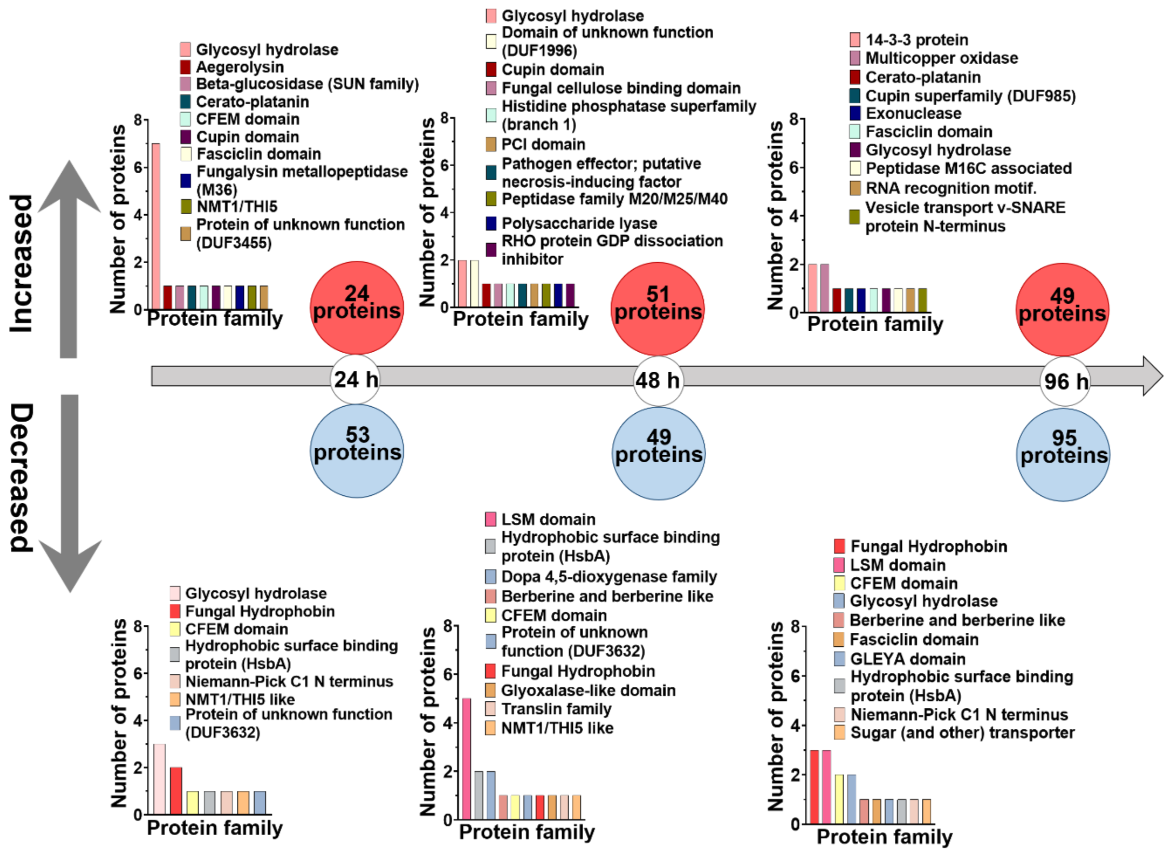

Figure S3. Protein family classification of *T. atroviride* proteins secreted in response to *Arabidopsis* at 24, 48 and 96 h of co-culture. Graphic representation for the *Arabidopsis* proteins differentially modulated (increased or decreased) according to protein family classification predicted by HMMER server [93]. Only proteins that were not predicted as enzymes according to EC nomenclature system are shown.

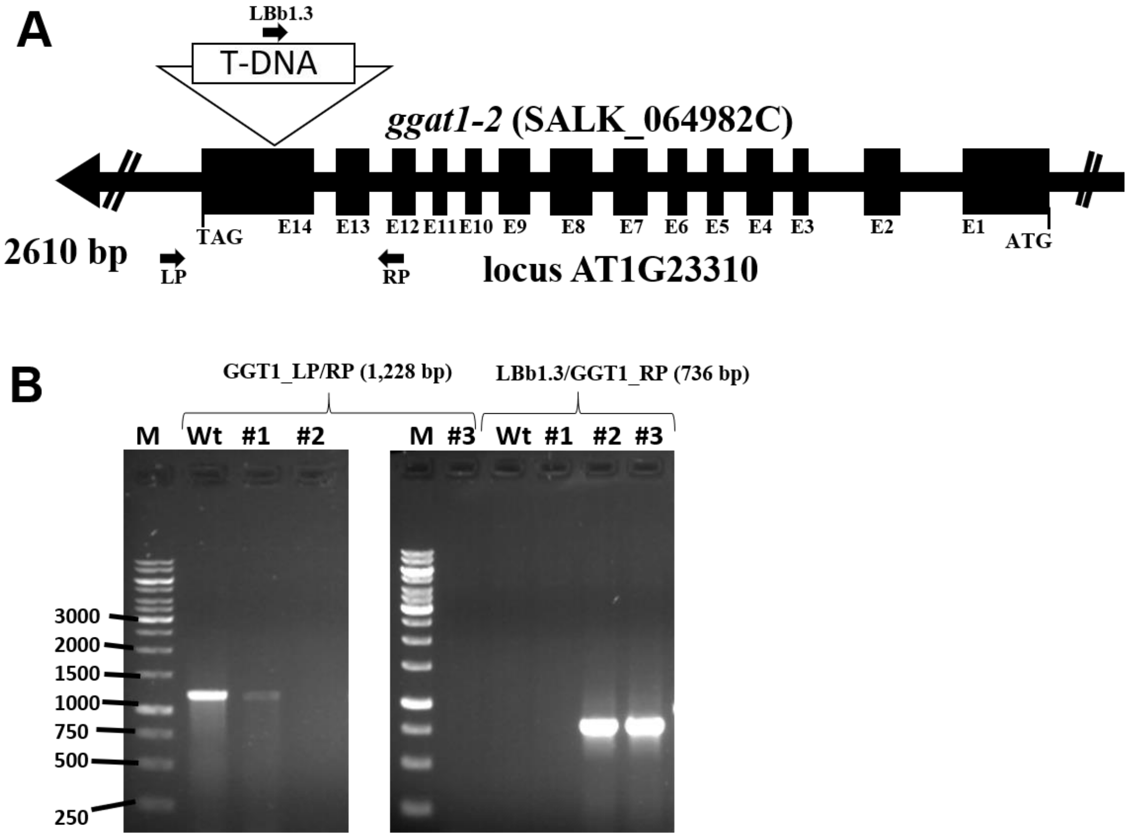

Figure S4. Genotyping of *ggat1-2* T-DNA insertional mutant. (**A**) Structure of the AT1G23310 locus. Primers used to screen the SALK_064982C line for homozygous mutants are indicated by black arrows RP, LBb1.3 and LP, which were designed using the SALK T_DNA primer design web tool (http://signal.salk.edu/tdnaprimers.2.html accessed on December 2018). E: Exon. (**B**) Genotyping PCR of *Arabidopsis* Col-0 (wt) plants and *ggat1-2* T-DNA insertional mutants. Bands at 736 bp in the right gel image indicate the presence of the T-DNA in the #2 and #3 plants corresponding to the *ggat1-2* mutant (using LBb1.3 and RP primers) whereas wt depicts a 1,228 bp band using LP and RP primers (left gel image). All primer sequences are listed in Table S3.

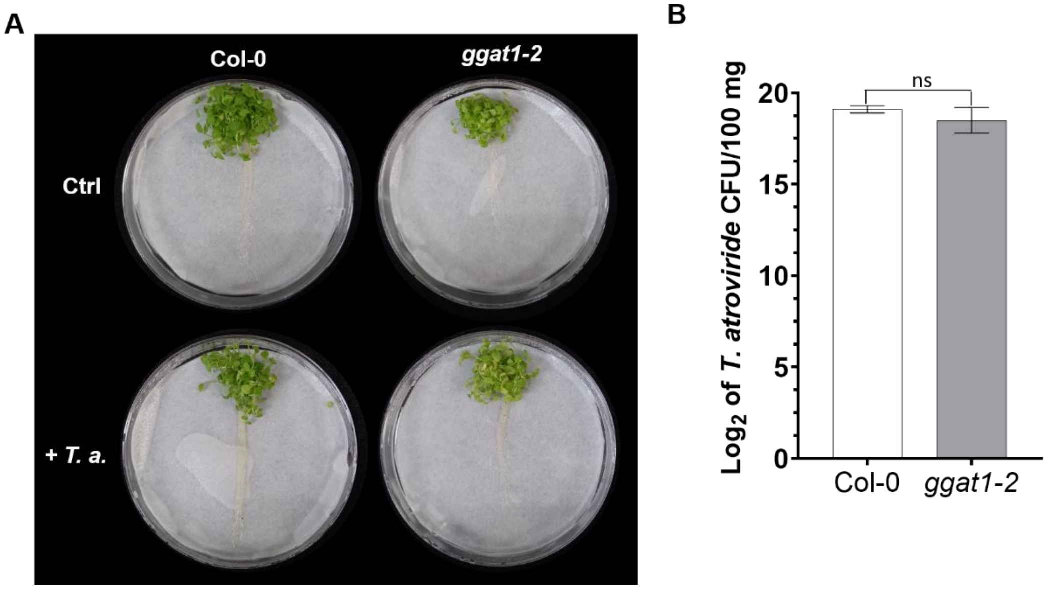

Figure S5. *Arabidopsis* root colonization by *T. atroviride* was similar for *ggat1-2* and Col-0 plants. *Arabidopsis* Col-0 and *ggat1-2* plants were grown for 15 days in 0.5 X MS medium and then their roots were immersed in a suspension of *Trichoderma* conidia (+ *T. a*). After 72 h, roots were surface sterilized with sodium hypochlorite, ground, and serial dilutions were plated on PDA selective medium. Log_2 of colony-forming units (CFU) were calculated per each 100 mg of roots. Control plants (Ctrl) were immersed in water and treated as above. Experiments were repeated twice with similar results. Results were analyzed using a Tukey multiple comparison test (α = 0. 05). N.S.: non-significant.

Table S1. Primers used in this study.

Gene name	TAIR locus number	Description		Sequence (5′ to 3′)
GGAT1	**At1g23310**	**Glutamate-glyoxylate aminotransferase 1**	**LP**	**TCTGCTTCTTCTGCGTTTAGG**
			**RP**	**AAAAACGTCGTGTGCAATTTC**
**LBb1.3**	**-**	**Left border primer for the T-DNA**		**ATTTTGCCGATTTCGGAAC**
